# Supplementary material for: Content-rich biological network constructed by mining PubMed abstracts
Source: BMC Bioinformatics. 2004 Oct 8;5:147. doi: 10.1186/1471-2105-5-147 (PMC528731; doi:10.1186/1471-2105-5-147)
Supplement: Additional File 5 — The original Chilibot query results of the term "long-term potentiation (LTP)" and 22 other terms, limiting the latest references analyzed to the years 1990, 1995, 2000, and 2004. [file 1471-2105-5-147-S5.bz2 › chilibotAdditionalFile5/ltp2000/html/CREB_PKA.html]

 


 **CREB** and **PKA** 
  
Found 160 abstracts in PubMed,  **30 abstracts were retrieved and analyzed**.  


---

 Search Google  |
 PDF files only 
|  EDU domain only 

---

**Interactive relationship** (e.g. stimulation, inhibition, etc)

- Like protein kinase A  **PKA**  stimulated wild type  **CREB** , the Tyr134Phe polypeptide was found to stimulate target gene expression via the Ser133 dependent recruitment of CBP and p300.  Ref: 10825195 Mol Cell Biol, 2000
- **CREB**  protein, once cotransfected with hD2 promoter construct and  **pKA**  in F9 teratocarcinoma cells, which are unresponsive to cAMP, was able to stimulate the hD2 gene transcription.  Ref: 10803591 Endocrinology, 2000
- In contrast, cAMP phosphorylated  **CREB**  via  **PKA**  directly but required a Rap1 ERK pathway to activate a component downstream of  **CREB**  phosphorylation and  **CREB**  binding protein recruitment.  Ref: 10950954 J Biol Chem, 2000
- Dexamethasone further enhanced FSK or PMA induced proENK mRNA expression, which was not correlated with the activation of AP 1 expression and  **CREB**  phosphorylation, suggesting that synergistic interaction of glucocorticoid with  **PKA**  or PKC pathway for the regulation of proENK mRNA expression appears to be mediated by other pathways rather than  **CREB**  and AP 1 families.  Ref: 11113530 Brain Res Mol Brain Res, 2000
- On the basis of these studies, it is proposed that  **PKA**  independent stimulatory actions of GLP 1 at RIP1 are mediated by bZIP transcription factors related in structure but not identical to  **CREB** .  Ref: 10909973 Diabetes, 2000
- the redox molecules differentially regulate NF kappaB, AP 1, and  **CREB**  activation induced by TNFalpha, PMA, and forskolin and by expression of signaling intermediate kinases, NIK, MEKK, and  **PKA**  in HEK293 cells.  Ref: 10903915 Biochem Biophys Res Commun, 2000
- One of the best characterized stimulus induced transcription factors, cyclic AMP response element CRE binding protein  **CREB** , activates transcription of target genes in response to a diverse array of stimuli, including peptide hormones, growth factors, and neuronal activity, that activate a variety of protein kinases including protein kinase A  **PKA** , pp90 ribosomal S6 kinase pp90RSK, and calcium calmodulin dependent protein kinases CaMKs corrected.  Ref: 10872467 Annu Rev Biochem, 1999
- The cAMP regulated transcription factor  **CREB**  undergoes conformational change in response to phosphorylation by  **PKA**  at Ser 133.  Ref: 10886376 Genes Cells, 2000
- Binding of cAMP to the regulatory subunit of cAMP dependent protein kinase  **PKA**  is an essential step for cAMP mediated signal transduction including phosphorylation of cAMP response element binding protein  **CREB** .  Ref: 10791102 Rinsho Shinkeigaku, 1999
- Specific blockade of  **PKA**  with RpcAMPS prevented the activation of  **CREB**  and also the comitogenic effect of FO as well as of NE.  Ref: 11054474 Cardiovasc Res, 2000
- The blunted beta adrenoceptor mediated  **PKA**  response in fibroblasts from patients with major depression is reflected in a significant reduction in the isoproterenol stimulated phosphorylation of the nuclear transcription factor  **CREB** .  Ref: 11099740 J Affect Disord, 2000

**Parallel relationship** (e.g. studied together, co-existance, homology, etc.)

- In contrast, the action of GLP 1 at the CRE was not blocked by cotransfection with M1  **CREB** , an isoform that lacks a consensus serine residue serving as substrate for  **PKA**  mediated phosphorylation.  Ref: 10909973 Diabetes, 2000
- The transcription factor  **CREB** , a potential substrate of both protein kinase A  **PKA**  and RSK 1, is phosphorylated in response to IL 1beta and cAMP in HepG2 cells.  Ref: 10965886 Endocrinology, 2000
- Alteration of cAMP mediated signal transduction in cerebral ischemia binding activity of  **PKA**  and phosphorylation of  **CREB**  .  Ref: 10791102 Rinsho Shinkeigaku, 1999
- To further study this phenomenon, a developmental characterization of these phosphorylation sites relative to  **PKA** , cAMP dependent response binding element and phosphorylated  **CREB**  was performed using samples from mouse brain.  Ref: 10965152 Dev Neurosci, 2000
- Taken together, these results show that the major hypophysiotropic factors stimulate POMC transcription through a signaling cascade that involves  **PKA**  and  **CREB** .  Ref: 11081181 Pituitary, 1998
- BACKGROUND Previous in vivo studies show that acute ethanol exposure sequentially increases protein kinase A  **PKA**  activity, the phosphorylation of the adenosine 3 5 cyclic monophosphate cAMP dependent transcription factor,  **CREB** , and finally proenkephalin gene expression.  Ref: 10923996 Alcohol Clin Exp Res, 2000
- These studies demonstrate that the molecular mechanism of NE action on the expression of the ANG gene in OK cells may be mediated via both the  **PKA**  and PKC signalling pathways and via the phosphorylation of  **CREB** .  Ref: 11108138 Mol Cell Biochem, 2000
- The low EC 50 value of 1 nM isoproterenol for activation of gene expression in stably transfected human fibroblasts appears to be a reflection of the amplification mechanism occurring via the beta adrenoceptor cyclic AMP  **PKA**   **CREB**  transduction cascade.  Ref: 11099740 J Affect Disord, 2000
- Its role in LTM may be related to the peak of  **PKA**  activity, and to the levels of its substrate, nuclear P  **CREB** , that have been described in a previous paper to occur at 0 and again at 3 h after training.  Ref: 10780835 Behav Pharmacol, 1999
- Our results indicate that  **CREB**  but not ATF1 can support basal promoter activity while both can robustly induce the promoter activity in response to co expression of the catalytic subunit of cAMP dependent protein kinase  **PKA** .  Ref: 11108136 Mol Cell Biochem, 2000
- In contrast with isoproterenol, ethanol exposure did not increase cAMP accumulation,  **PKA**  activity, or the phosphorylated form of  **CREB** .  Ref: 10923996 Alcohol Clin Exp Res, 2000
- In contrast to monocytic cells, in T cells cAMP induced  **PKA**  dependent phosphorylation of the CRE binding protein 1  **CREB**  1 seems to play a marginal role in IL 10 induction, which was reflected by a low cAMP dependent IL 10 promoter enhancer stimulation in reporter gene assays.  Ref: 11108135 Mol Cell Biochem, 2000
- In the present study, binding activity of  **PKA**  with cAMP and  **CREB**  phosphorylation were examined in rat focal brain ischemia induced by occlusion of the middle cerebral artery for 1.5 hours followed by various time of recirculation.  Ref: 10791102 Rinsho Shinkeigaku, 1999
- There was no developmental change in the time course of phospho  **CREB**  induction that followed KCl depolarization or  **PKA**  activation, nor was there a developmental change in the time course of phospho Erk1 2 induced by NMDA receptor activation.  Ref: 10804193 J Neurosci, 2000
- The detection of spatial novelty is associated with a sequential activation of  **PKA** , ERKs p44 and p42 MAPKs and CaMKII and the phosphorylation of c AMP responsive element binding protein  **CREB**  in the hippocampus.  Ref: 11040265 Learn MemLearn Mem, 2000
- Calcium mediated phosphorylation of  **CREB**  through the  **PKA**  Rap1 ERK pathway.  Ref: 10950954 J Biol Chem, 2000
- We conclude that differentiation dependent transcription of the CGalpha gene in villous trophoblasts is mainly governed by increasing expression of AP 2alpha and  **PKA**  dependent phosphorylation of  **CREB**  1 and ATF 1.  Ref: 11014230 Endocrinology, 2000
- Multiple signaling pathways have been shown to contribute to the regulation of  **CREB**  dependent transcription, including both protein kinase A  **PKA**  and mitogen activated protein MAP kinase extracellular signal regulated kinase ERK dependent kinase cascades.  Ref: 10950954 J Biol Chem, 2000
- BACKGROUND The second messenger cAMP stimulates the expression of numerous genes through the  **PKA**  dependent phosphorylation of  **CREB** .  Ref: 10886376 Genes Cells, 2000
- This effect was not due to activation of any of the previously known  **CREB**  kinases, including  **PKA** , CaMK, PKC, p70s6k, or MAPKAP kinase 2.  Ref: 10849656 Adv Exp Med Biol, 2000
- This switch in isoform composition just precedes the change in the extent of phosphorylation at serines 214 and 409, and occurs at a time when  **PKA**  phosphorylation of  **CREB**  is increasing.  Ref: 10965152 Dev Neurosci, 2000
